# Employers’ requests when advertising for nurses—A national mapping of recruitment advertising for nurses in Sweden

**DOI:** 10.1371/journal.pone.0303255

**Published:** 2024-07-12

**Authors:** Camilla Fröjd, Eva Jangland, Anna-Karin Gunnarsson

**Affiliations:** Department of Surgical Sciences, Uppsala University, Uppsala, Sweden; Alexandria University Faculty of Nursing, EGYPT

## Abstract

**Introduction:**

There is a shortage of nurses and many are leaving the profession. Maintaining sufficient nursing staff is a major healthcare challenge for societies worldwide. Work conditions, job orientation, and career opportunities all factor into nurses’ rates of attrition, exit, and turnover. Newly graduated nurses have requested structured introductory and/or mentoring programmes to ease their transition from education to work life and develop the skills and knowledge necessary in their particular work setting. Nurses also seek opportunities to continue learning and developing professionally.

**Aims:**

To map and describe the content of recruitment advertisements for nurses. Research questions were: ‘What qualifications do healthcare employers request when recruiting nurses?’ and ‘What sorts of professional development do healthcare employers offer nurses?

**Materials and methods:**

A comprehensive national mapping of recruitment advertisements for nurses in Sweden where all advertisements, N = 450, on 20 of the 21 regional hospital websites were collected. A qualitative and a quantitative content analysis was performed.

**Results:**

Personal characteristics dominated requested competence in recruitment advertisements. Employers offered general nursing opportunities with unspecific work content and focused more on recruiting newly registered, rather than experienced, nurses. In only a few advertisements, employers asked for a master’s degree. No employer requested nurses with a PhD or research experience.

**Conclusion:**

While the World Health Organization stresses the need for a sustainable recruitment and attainment of nurses to secure health care, employers’ recruitment of mostly newly graduated nurses and offering little professional development and few career opportunities may be one explanation for the difficulties in securing safe nurse staffing.

## Introduction

Maintaining sufficient nursing staff is a major healthcare challenge for societies worldwide and at the same time there is a global shortage of nurses. The World Health Organization (WHO) estimates that an additional 9 million nurses and midwives will be needed by 2030 [1]. In Sweden, Eighteen of twenty-one regions report shortage of registered nurses and 21/21 regions shortage of postgraduate nurses [2]. Healthcare systems can function only with access to and retention of competent, motivated, and empowered health workers. WHO has developed a global human resources strategy aimed to ensure the universal availability, accessibility, coverage, and quality of healthcare workforces by 2030 [1]. Similarly, the International Council of Nurses (ICN) concludes that safe staffing (having enough nurses with adequate skills, education, and experience available at all times across the continuum of care) is critical to patient safety and quality of care [3].

High attrition, exit rates, and turnover in nurses make recruiting and retaining nurses an international challenge. Attrition rates for healthcare workers vary between from 3% to 28%, with average voluntary attrition at 7% for nurses [4, 5]. Intention-to-leave among nurses varies from 5% to 30% in Europe [5, 6]. A longitudinal project showed that in 2002, after only one year of qualifying, 10% of Swedish RNs planned to leave the profession; by 2006, that number had increased to 20% [7]. At a follow-up five years after graduation, 20% of the remaining nurses stated that they intended to leave the profession.

Nurse turnover involves high costs that are largely avoidable. Although different methods of calculating costs and defining turnover make between-country comparisons difficult, a review showed that costs in Australia, New Zealand, the USA, and Canada ranged from about US$20,000 to US$48,000 (€17,000–40,000) per turnover [8, 9]. In Sweden, The Swedish association of Health Professionals [10], has estimated a cost range of US$11,000 to $34,000 (€9600–€28,000) per turnover.

Work conditions, job orientation, and career opportunities all factor into nurses’ rates of attrition, exit, and turnover [11]. In a Canadian hospital, improved work conditions, including full-time work and extended periods of orientation and mentorship, reduced turnover from 10% to 1% in newly graduated nurses’ first 3 months at work, and from 26% to 5% in their first year [12]. In a recent study the results showed that almost half of the nurses found it difficult to adapt to nursing and about half of the nurses planned to leave their institutions [13]. Settings with insufficient nursing staff are associated with emotional exhaustion and work dissatisfaction [14], which could cause even more nurses to leave [15, 16]. Newly graduated nurses request structured introductory and/or mentoring programmes to ease their transition from education to work life and develop the skills and knowledge necessary in their particular work setting [17–21]. Nurses also seek a healthy work/life balance, recognition, adequate supervision [6, 22, 23], and opportunities to continue learning and developing professionally [5, 16, 24, 25]. In order for new nurses to develop their clinical gaze, new nurses need opportunities to reflect and learn from experienced colleagues and this must be incorporated as natural parts of the daily work [26]. Other studies on ‘Generation Y’ nurses show that nurses want to specialize or continue studying during their careers [18, 22]. Providing career opportunities and making good use of nurses’ competencies may reduce turnover [18, 24] while a lack of such opportunities may lead to resignations [27]. The importance of recruiting and retaining qualified and experienced nurses has been demonstrated in several studies showing that higher academic nursing qualifications correlate to lower patient mortality and higher quality of care [14, 28, 29]. To recruit and retain competent nurses, healthcare organizations need to meet the needs and expectations of both newly graduated and experienced nurses.

Employers struggle to recruit and retain the qualified and experienced nurses who are crucial to healthcare organizations. Recruitment advertisements (ads) for nurses should specify the content and associated professional benefits of the job, such as orientation and opportunities for professional development, as well as the required qualifications and work experience. A national mapping of the content of recruitment ads should therefore provide a comprehensive overview of the work content and opportunities for career and professional development offered to nurses in Swedish health care. To the best of our knowledge, this is the first comprehensive national mapping of recruitment ads for nurses in Sweden.

### Aims

We aimed to map and describe the content of recruitment advertisements for nurses throughout Sweden by asking the following research questions: ‘What qualifications do healthcare employers request when recruiting nurses?’ and ‘What sorts of professional development do healthcare employers offer nurses?

## Materials and methods

The study was a national mapping of recruitment ads for nurses in Sweden. Registered nurses (‘nurses’ in this paper) belong to a regulated profession that requires a Bachelor of Nursing or Caring Sciences degree attained within a 3-year vocational nursing programme. Nurses have an autonomous responsibility for nursing care in the Swedish healthcare system. To ensure rigour in the study, we followed the consolidated criteria for reporting qualitative research guidelines [30].

### Study design

This is a qualitative study with an inductive approach using nurse recruitment ads which enables an understanding of how nurses are recruited in Sweden.

### Setting

The context for this study is hospital care, including all medical specialities. Swedish taxes fund the healthcare system, which is organized into 21 regions. Hospitals usually recruit nurses via ads on their websites. Nurses work either directly for a hospital or by contract through a private staffing company providing nurses on an as-needed basis.

Postgraduate specialist diplomas in nursing are available in medical, surgical, intensive, perioperative, prehospital, paediatric, oncological, psychiatric, geriatric, and anaesthetic care, and new nursing specialities continue to emerge (e.g., in emergency and diabetes care). These specialist degrees are one-year professional post-baccalaureate qualifications that include an examination at the master’s level. Nurses may also complete a master’s programme for advanced practice in nursing leading to employment as a nurse practitioner [31] and academic degrees at the master’s or PhD level. Since Midwifery is a protected professional title, midwives are excluded from this study.

### Sample

All ads that were available in May 2016 on 20 of the 21 regional hospital websites were collected in May 2016, excluding one region by mistake. Inclusion criteria stipulated ads recruiting nurses for inpatient care and all specialties within somatic, psychiatric, and prehospital care (N = 450). We excluded all ads for managerial, rather than hands-on, nursing positions. All ads were in Swedish. All ads were printed, filed and the analysis was performed using the printed ads in paper form. The length of the ads in total varied between 100–900 words, and formalities and other information not relevant to the aim of this study were included. Only text describing qualifications and professional development formed the basis for the calculation of the number of words.

### Data collection

We collected the ads (N = 450) in May 2016. In October 2020 we conducted a follow-up in 4 of the 21 regions (randomly selected) to be compared to the content of nursing recruitment ads over time. All newly selected recruitment ads, (N = 102), from 13 hospitals in the 4 regions followed the original inclusion criteria, and a comparison showed no differences in their content to those collected in 2016.

### Data analysis

Inductive content analysis was used [29]. The first and last author read through the ads to become familiar with the material as a whole. The last author performed the open coding, compared the codes and classified them into subcategories based on their differences and similarities. The last author suggested categories. The first, second and last author then read the subcategories and categories and all authors discussed subcategories and categories until agreement regarding the categorisation was achieved. The first and last author then chose citations from different hospitals and ads to be used to clarify and concretize our results. An example of the process of the analysis is presented in Table 1.

**Table 1:** Process of the analysis.

Code	Subcategories	Category
Have experience	General competence	Competence
Postgraduate education	Formal competence	

To explore the extent to which employers ask for formal qualifications and offer professional development, we performed a quantitative content analysis where the last author counted the number of words relevant to the research questions in each subcategory. We then calculated the percentages of relevant words to find the proportions of each category in the total data.

We excluded the following prerequisites for work as a registered nurse from the analysis: nursing licence, driving licence for work in prehospital care, and postgraduate diplomas for work in anaesthesiologic, operating theatre, or intensive care since they are all mandatory.

### Ethical considerations

According to national ethical directives in Sweden, studies not collecting data related to health, sensitive personal data or sexual, political or religious orientation do not require approval by an ethical committee [32]. Data consists of recruitment advertisements and no humans have provided data. The research was conducted in accordance with ethical standards. All available ads were included in the study and we present the results at a group level and do not identify any individual recruitment ad, hospital, or region.

## Results

Results are presented in four categories including 11 subcategories. In answer to the research question of what qualifications healthcare employers request when recruiting nurses, two categories emerged: *Competence* and *Personal Characteristics*. The subcategories within Competence were *formal competence*, *academic merits*, *experience in leadership and/or teaching* and *general competence*. Personal Characteristics consists of *personal traits*. In answer to the research question of what sorts of professional development healthcare employers offer nurses, two categories emerged: *Career Opportunities* and *Content of Work*. The subcategories of Career Opportunities were *introduction*, *specific career opportunitie*s, *general career opportunities*, and *research*. The subcategories of Content of Work were *leadership*, *teaching*, *development*, and *general content* (Table 2).

**Table 2:** Employers’ requested qualifications and offers of professional development: Subcategories with codes in brackets to exemplify the textual content of the ads, and categories.

Qualifications requested by employers		Professional development offered by employers	
Categories		Categories	
Competence	Personal characteristics	Career opportunities	Content of work
Subcategories	Subcategories	Subcategories	Subcategories
Formal competence (Postgraduate education, courses, special skills)	Personal traits (Positivity, flexibility, calmness, communication skills)	Introduction (Individual introduction, trainee programmes)	Leadership, teaching, and development (Teaching students, developing care)
Academic merit (Master’s degree)		Specific career opportunities (Courses, post graduate diplomas)	General content of work (Coordination [‘spider in the web’], cooperation, responsibility)
Experience of leadership, teaching, and development (Teaching students, leading a team)		Research (PhD candidacy)	
General competence (Experience, interest)		General career opportunities (Continuous development, individual development)	

### Qualifications requested by employers

#### Competence

*Formal competence*. In this subcategory, some employers requested, but did not require, nurses to have a specialist diploma, and some requested newly graduated nurses and experienced nurses in the same sentence. Employers in this subcategory asked for nurses with experience, skills, and/or education specific to the job. Competences ranged from excellent computer skills to specialized education or experience. A common request was for experience in the advertised speciality. Some employers also wanted nurses to have knowledge of a certain system for medical records or to be educated in specialties such as psychotherapy or the care of patients with asthma or diabetes.

*Academic merit* formed a subcategory. Having a master’s degree was mentioned only three times in the ads, all related to combined university/hospital positions at one university hospital in Sweden. None of the 450 recruitment ads requested a bachelor’s degree, PhD or any research experience.

*Experience in leadership*, *teaching*, *and/or development*, formed a subcategory only where employers were trying to fill special positions such as being an assistant manager/team leader combined with working as a nurse or jobs where working as a nurse was connected to a university.

*General competence*. In this subcategory employers asked for general competence by using words that were neither specific to the job nor descriptive of a specific competence, such as ‘work/life experience’ (Ha 1) or ‘good knowledge of nursing and health care’ (V1). Employers asked for nurses with an interest in nursing or certain medical aspects, experience working as a nurse, comfort in the professional role, autonomy, or the ability to handle stressful situations. In several ads employers asked for experience, without any more specific definition of what experience would interest them.

#### Personal characteristics

*Personal traits*. In the subcategory the employers expressed a preference for nurses who were service-minded, positive, flexible, stress-resistant, or able to see the opportunities within changes at the workplace. Employers expressed that they sought nurses who were calm and dedicated, liked variation in the pace of the work and some employers expressed that they sought nurses who were more interested in the work than in gaining prestige. Some employers asked for several different traits at once: ‘You always put the patient first, you have high accountability and you are highly motivated in your work. This shows not only in your high work morale but also in your fearless and determined personality’ (S1). Employers were looking for nurses who were good communicators, took responsibility for their work tasks, and were able to plan, organize, and prioritize their work. The nurses needed to be team players, cooperative, and empathetic. Nurses also needed to be autonomous in their work and able to make good decisions quickly, handle acute and complex situations, delegate, and maintain focus on the patient.

One employer described the desired personal characteristics as follows: ´Great importance will be put upon personal characteristics such as a will to take on responsibility, cooperating skills and the ability to handle unexpected and complex situations. You must be able to respond well to patients with dementia in a professional manner. You are interested in developing and improving the work place. You possess good self-knowledge, are professional and committed and with a huge interest in working with elderly persons`(St 1).

### Professional development offered by employers

#### Career opportunities

*Introduction*. Employers frequently offered new employees an individual introduction to the job, which could be based on needs or structured. Some employers also offered introductory or training programmes and others also offered mentoring in this subcategory.

*Specific career opportunities*. In this subcategory employers offered a range of opportunities from courses relevant to the job to postgraduate diplomas in specialist nursing. For example, one ad said ‘We have generous terms when it comes to supporting postgraduate diplomas in Specialist nursing’ (Vb 1).

*General career opportunities*. Employers commonly offered nurses a chance to continue developing their skills and competence in this subcategory. One ad offered nurses ‘individual professional development should you not find the day-to-day work challenging enough’ (St 2).

*Research*. Only four ads from one region offered nurses the opportunity to conduct their own research.

#### Content of work

*Leadership*, *teaching*, *and development*. Work advertised in this subcategory included leading the team or the day-to-day work, teaching students in clinical practice, teaching patients self-care before discharge, and developing or improving the work and/or nursing care.

*General content of work*. Some ads in this subcategory asked for nurses to be the spider in the web, to make sure of patients’ needs being met, or to adjust the work according to their interests and competence. Work content described in terms of working in teams close to the patient or in cooperation on the ward, at the clinic, at the hospital, and/or with external health care partners. The content of work mentioned in these ads also included planning and performing the work, working with modern methods, or taking responsibility for achieving high patient safety, as in this example: ‘We offer you an interesting job and development as a nurse in keeping with our values of responsibility, humanity, and a holistic view [of patient care]’ (St 3).

### Quantitative analysis of qualifications requested and professional development offered by employers

In total, 4560 words relevant to the research questions were identified in the data analysis, 2997 words for qualifications requested and 1563 words for offers of professional development (Table 3).

**Table 3:** Number of words relevant to qualifications requested by employers and professional development offered by employers, and proportions of words between the categories presented by percentage.

Qualifications requested by employers (2997 words)		Professional development offered by employers (1563 words)	
Competence n = 630 (21%)	Personal characteristics n = 2367 (79%)	Career opportunities n = 427 (27%)	Content of work n = 1136 (73%)

Personal characteristics contributed 79% of words about requested qualifications, and other competences contributed 21%. Ads offering professional development expressed content of work in 73% of relevant words and 27% were career opportunities. Personal traits, nonspecific career opportunities, and nonspecific content of work dominated the content of the ads (Table 3).

## Discussion

This study aimed to map and describe the content of recruitment advertisements for nurses. The research questions we set out to answer concerned what qualifications healthcare employers request when recruiting nurses and what sorts of professional development healthcare employers offer nurses.

The results reveal a national structure in which nurses were systematically recruited for their personal traits rather than their competence. Employers usually offered general nursing opportunities with unspecific work content and focused more on recruiting newly registered, rather than experienced, nurses. In only a few ads, employers asked for a master’s degree, and opportunities for PhD candidates appeared in only four ads, all for the same university hospital. No health care employer requested nurses with a PhD or any research experience.

Personal characteristics dominated competence in recruiting ads for nurses. Personal characteristics, however, are not professional skills and the personal characteristics requested are neither related to nor specific to professional nurses or nursing in general. While employers may need to seek some specific personal characteristics in new recruits, the absence of requests for specific competencies, education, or postgraduate nursing diplomas was widespread and notable. Furthermore, when employers did ask for specific experience, education, or a specialist nurse degree, these competencies were not always mandatory and were often expressed ‘desired’ or ‘meritorious’.

The evidence of the relationship between nurses’ higher formal competence and better patient outcome is increasingly recognised worldwide [28, 33]. Based on this research, it would be reasonable to expect that ads for nurses should include requests for formal competence and academic merits. The absence of concern about these qualification raises questions about how health care employers view and value nurses’ knowledge and formal competence.

No employers asked for nurses with a PhD degree. Our experience is that some nurse managers believe that ‘not every nurse wants to be a researcher.’ While this may be true, even if not all nurses want to be researchers or pursue a career in nursing, establishing structured professional development and clinical positions related to formal competence and academic merit will likely retain the most ambitious nurses, who then can serve as mentors and senior nurses to their newer colleagues. Over 1700 nurses/midwives in Sweden have a PhD [34] and that number is growing. It is impossible to obtain comprehensive data about how many of these nurses/midwives are employed in health care or what positions they have since Swedish health care registers do not track nurses’ degrees or positions. Following collection of the present data, a review published in 2021 verified that few clinical positions exist where nurses with a PhD can contribute to evidence-based practice and improve care [35]. To change this, nurse managers need to create conditions for evidence-based practice, including well-defined clinical roles for nurses with a PhD [36]. The absence of structures for developing nursing through research, and offering professional development with or without conducting research likely contribute to why nurses have difficulty seeing a future in Swedish health care.

Offers of professional development in the ads collected consisted mainly of introductory programmes, in line with research showing that orientation and mentoring programmes are beneficial for successfully recruiting newly graduated nurses [17, 19, 20, 37, 38]. On the positive side, this is in line with studies showing the importance of providing new nurses with good conditions such as opportunities to reflect, learn and have support from experienced colleagues in order to make a smooth transition into the nurse role [39–42]. This transition is stressful for new nurses and there is a risk that without experienced colleagues, new nurses choose to leave. As Källestedt et al. points out, such good conditions for transition into being a nurse need to be an integrated part of daily work [26]. To achieve this, one would wish that employers would focus perhaps a bit more on recruiting as well as retaining experienced nurses and include them in a work structure where reflection and knowledge transfer can occur. We believe that this could also serve a meaningful opportunity for personal growth which also most likely could decrease nurse turnover.

Although research shows that professional development is an important factor in retaining nurses [34, 38, 43, 44], opportunities for professional development and career opportunities were mostly nonspecific. This could be interpreted to mean employers lack effective strategies to build and maintain adequate nurse staffing and use nurses’ competencies appropriately. It could also be the consequence of a downward spiral in which high nurse turnover causes employers to switch into ‘panic-mode’ and focus on recruiting newly graduated nurses instead of building long-term strategies of using professional development to retain nurses and utilize their competence. Such long-term strategies could support knowledge transfer and achieve stability in nursing staffing, which in turn could improve the work environment and increase retainment of nurses over time.

Instead, recruiting experienced nurses, and offering them a challenging career, could benefit the organization in several ways. Experienced nurses could provide stability in staffing levels by reducing stress on the ward through helping to retain, supervise, mentor, and educate newer nurses; improve patient outcomes and quality of care; and continue to develop nursing practice. Employers should therefore express their interest in recruiting such highly qualified nurses in addition to new graduates, ask for specific competences such as academic degrees and specialist education, and offer them clear career paths and further professional development.

### Strengths and limitations

The study is strengthened by the nationwide data collection. This approach gave rich data to the results and we assess that all possible variations in the content of nurse recruitment ads are represented in the results. Trustworthiness was considered throughout the study. The authors of this paper have extensive knowledge of nursing from both clinical and educational perspectives, and experience in strategic staffing. Confirmability was enhanced as the authors were involved in the analysis, with multiple meetings discussing the findings until consensus were reached. To strengthen credibility, citations were discussed and added to support the findings. Data were collected at two different time points. The first data collection took place in 2016. Although it could be a limitation that seven years now have passed since then, the data collection was comprehensive and nationwide with all ads from 20 of 21 regions in Sweden. We would like to suggest that the results are strengthened by the second data collection in 2020 since the content of the ads in the second data collection were similar to those in the initial data collection. One interpretation is that neither the Swedish healthcare system nor employers have changed their strategies to recruit and retain nurses over the four years passing between the two data collections. The similarity of content at the two data collections strengthens our assumption that our results may well reflect the reality in which healthcare employers recruit nurses in Sweden.

It was unfortunate to miss one of the regions, however we are convinced that this does not affect the results to any extent since Swedish health care is homogenous and there are small, if any differences regarding nurse recruitment and staffing.

A limitation could be that, despite the randomized selection, we missed ads requiring a PhD or research experience in the second data collection. However, it is reasonable to conclude that there are still few research positions available in Swedish health care. This is an important finding strengthening our interpretation that the lack of clinical research positions for nurses is ongoing and systemic.

Albeit unusual to add a quantitative data analysis to a qualitative paper, analysing the proportions of what employers seek from nurses and what they offer them is relevant to understanding the systemic biases and trends in Swedish nurse recruitment. Recruitment ads reflect healthcare employers’ priorities and their views on nurses.

The present study shows the content of nurse recruitment ads, but it does not explain the results. We believe that analysis from a wider societal and historical perspective is needed to explain the continuing emphasis in Swedish health care on hiring nurses for their personal characteristics over their academic qualifications and research experience.

Whether or not the results of this study are transferable to different nations is unknown. However, this investigation of the structure of nursing recruitment in one country may be an important starting point in supporting and empowering nurses to work at the forefront of progress in health care.

## Conclusion

While the World Health Organization stresses the need for a sustainable recruitment and attainment of nurses to secure health care, employers’ recruitment of mostly newly graduated nurses and offering little professional development and few career opportunities may explain the difficulties to secure safe nurse staffing.
